# Diagnostic Quandary of Granulomatosis With Polyangiitis Presenting First in the Petrous Apex

**DOI:** 10.1155/crot/8773843

**Published:** 2025-07-28

**Authors:** Michael S. Castle, Matthew M. Carter, Alexander Poulakis, Li-Xing Man, Isaac L. Schmale

**Affiliations:** ^1^Department of Otolaryngology Head and Neck Surgery, University of Rochester Medical Center, Rochester, New York, USA; ^2^University of Rochester School of Medicine and Dentistry, University of Rochester Medical Center, Rochester, New York, USA

**Keywords:** granulomatosis with polyangiitis, lateral skull base, mastoiditis, petrous apex, petrous apicitis

## Abstract

**Objectives:** To describe a rare case of granulomatosis with polyangiitis (GPA) initially presenting at the petrous apex, accompanied by a brief literature review.

**Methods:** A detailed retrospective single-case study of a 29-year-old male diagnosed with GPA. A review of the scientific literature of GPA affecting the petrous apex, causing cranial neuropathies and/or ottorhrea was conducted.

**Results:** A 29-year-old male with a history of schizophrenia presented with right-sided otalgia, initially diagnosed as acute otitis media. Despite antibiotics, his symptoms persisted. Weeks later, he returned with cranial neuropathies and clear otorrhea. Imaging findings lead to a working diagnosis of skull base osteomyelitis despite noninfectious signs. His course was further complicated by his discharge against medical advice. Eventually, he was found to have a septal perforation and diffuse nasal inflammation. An autoimmune workup revealed c-ANCA-positive GPA. Subsequent kidney biopsy confirmed GPA, and appropriate therapy led to clinical improvement and near-complete resolution of skull base inflammation.

**Conclusions:** This case underscores the diagnostic complexity of GPA. Initial misdiagnosis of infection delayed appropriate treatment. Awareness of the varied presentations of GPA, including rare manifestations like skull base involvement and symptoms such as otorrhea and conductive hearing loss, is valuable. Early consideration of autoimmune etiologies and timely serological and histopathological analyses can prevent diagnostic delays and unnecessary treatments, improving patient outcomes.

## 1. Introduction

Granulomatosis with polyangiitis (GPA), formerly known as Wegener's granulomatosis, is an autoimmune vascular disease of medium and small-sized blood vessels that results in necrotizing granulomas typically affecting the paranasal sinuses, lungs, and kidneys [1]. However, GPA has also been reported to manifest in many other organ systems, such as the ears, the central nervous system, and various aspects of the skull base, albeit much less frequently [2]. Given its nonspecific initial presentation with elevated inflammatory markers and variable imaging characteristics, GPA may be mistaken for other inflammatory or infectious diagnoses, resulting in a delay in diagnosis. We present a case of a 29-year-old male with GPA who presented with otologic symptoms and primary skull base involvement on computed tomography (CT) and magnetic resonance imaging (MRI) suggestive of a skull base infection.

## 2. Case Report

A 29-year-old male with a history of schizophrenia first presented for right-sided otalgia to his primary care office. On initial examination (Week 1), his tympanic membrane was erythematous with a middle ear effusion, and he was diagnosed with acute otitis media and prescribed oral antibiotics. One week later (Week 2), he was seen for follow-up by a different provider at his primary care and was prescribed a new antibiotic for persistent symptoms and exam findings. Four weeks later (Week 6), he presented to the emergency department (ED) with a right-sided headache, and a CT scan of his head showed that his right mastoid was completely fluid-filled. Before this could be evaluated further, the patient left against medical advice. He was then seen by otolaryngology at another facility 2 weeks after (Week 8), where physical exam showed clear fluid abutting and therefore partially obstructing the right tympanic membrane. He reported sustaining head trauma during an assault sometime prior to his ongoing work-up, which, combined with the recent findings on CT, raised new concern for temporal bone fracture with possible cerebrospinal fluid (CSF) leak and led to recommendation to report to the local university hospital for otolaryngology and neurosurgery consultation.

**Figure 1:**
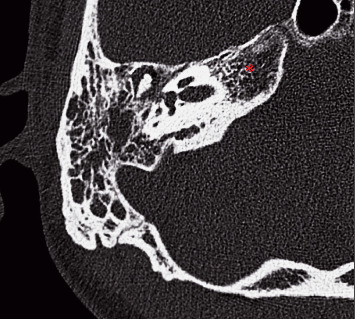
CT temporal bone without contrast in the axial plane with bone windowing. This scan shows mastoid opacification including opacification of air cells in the petrous apex highlighted with the asterisk (^∗^).

**Figure 2:**
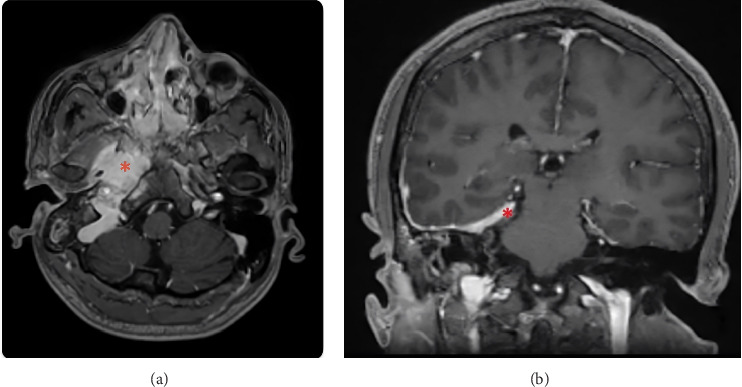
(a): Axial view of a T1 MRI with contrast showing enhancement along the right mastoid and petrous apex (highlighted with the asterisk (^∗^)). This was interpreted as concerning for mastoiditis and osteomyelitis. (b): Coronal view of a T1 MRI with contrast prior to treatment demonstrating dural thickening and enhancement along the skull base (asterisk (^∗^)), petrous apex, and mastoid.

**Figure 3:**
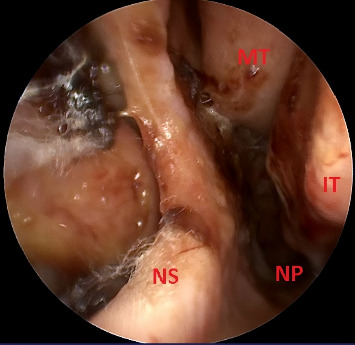
Nasal endoscopy of the left nasal cavity showing a large septal perforation and nasal inflammation with significant nasal crusting. NS: nasal septum, NP: nasopharynx, IT: inferior turbinate.

He presented to the ED at the university hospital that same day with intermittent headache, right-sided clear otorrhea, and facial numbness. He was afebrile with normal vital signs and not ill-appearing. He had right-sided facial numbness in a V2/V3 distribution and weakness of the right marginal mandibular nerve. Initial examination of the right external auditory canal showed clear fluid that filled the canal, which, combined with edema of the external auditory canal, obstructed the view of the tympanic membrane. Labs were notable for an elevated erythrocyte sedimentation rate and c-reactive protein, but an unremarkable leukocyte count and negative beta-2 transferrin test (which ruled out CSF leak). While admitted, a CT of the temporal bones demonstrated complete opacification of the right mastoid air cells without periosteal abscess or lateral skull base defect (Figure 1). Subsequent MRI revealed enhancement of the right petrous apex with surrounding inflammatory dural involvement (Figures 2(a), 2(b)). Subtle asymmetry of the right Eustachian tube prompted a nasal endoscopy, which showed a large septal perforation, adenoid hypertrophy, and diffuse nasal inflammation with crusting (Figure 3). Given these initial findings, and concern for infection, the primary team treated with intravenous antibiotics for presumptive skull base osteomyelitis. Before receiving further workup, the patient again left against medical advice, but accepted oral antibiotics and follow-up.

One week after discharge (week 9), he visited our neurotology clinic with reported improvement of his otorrhea and otalgia but persistent hearing loss. His physical exam was mostly reassuring, without otorrhea, tympanic perforation, or signs of infection, but was notable for moderate-to-severe conductive hearing loss. Despite a leading diagnosis of ossicular discontinuity, he refused middle ear exploration and possible ossicular chain reconstruction.

Approximately 2 weeks after last evaluation (Week 11), he presented to the ED for ankle swelling and left lower extremity edema. He continued to endorse hearing loss. A cavitary lung lesion was incidentally found on CT angiogram while ruling out pulmonary embolism. Tuberculosis workup was negative on readmission. Repeat CT/MRI revealed similar findings in the petrous apex, which were reported as concerning for an infectious etiology despite normal temperature and leukocyte count at that time. He was started on amoxicillin–clavulanate by infectious disease while a broader workup was pursued. He was found to be positive for c-ANCA, PR3, and proteinuria and negative for myeloperoxidase. A subsequent kidney biopsy revealed findings consistent with GPA. He was started on appropriate therapy and improved clinically, with repeat MRI of the head after 1 month of treatment (Week 15) demonstrating a near complete resolution in tissue inflammation and enhancement of the petrous apex.

## 3. Discussion

Here, we present a male with GPA who presented with nonspecific clinical and radiographic findings leading to a treatment delay due to primary initial skull base involvement with concern for osteomyelitis. Imaging of his skull base and elevated inflammatory markers initially led experienced clinicians of multiple specialties astray. His workup was further complicated by his psychiatric illness and leaving against medical advice. Ultimately, he developed a more classic presentation of GPA (nasal and kidney involvement), but his initial symptoms included trigeminal hypesthesia, otorrhea, and conductive hearing loss which are uncommon GPA findings contributed to diagnostic uncertainty. During his initial hospitalization, a septal perforation and increased nasal crusting were documented but overlooked given the concern for skull base infection. Also, on previous encounters, these findings were not observed. In hindsight, these rhinologic findings and a high index of suspicion could have led to an expedited diagnosis, but also a knowledge of the less common manifestations of GPA perhaps would have expedited his care.

Although head and neck involvement is common in GPA, skull base findings have been rarely documented. The largest study to date of GPA patients with skull base involvement was by Kiessling et al. and assessed 29 patients over a 22 years period [1]. This study reported trigeminal involvement in a majority of the cohort's initial presentations, like our patient, but no instances of otorrhea. Hearing loss has been well-documented as a potential presentation of GPA, but otorrhea has only a handful of reports, such as by Koenen et al. [3]. In their report, the patient underwent tympanostomy tube placement, and initial concern for an ear infection clouded the diagnostic picture prior to GPA treatment. Similarly, the presence of otorrhea in our patient was a factor that incorrectly led to an infectious etiology being pursued and served as a red herring. A recent systematic review described otorrhea being slightly more common, seen in 21% of patients in their series of 28 patients, but we would be remiss to overlook that this still represents only 6 reported cases of otorrhea in the setting of GPA with skull base involvement [4]. This case demonstrates the importance of considering autoimmune workup with suspicion for GPA in patients presenting with skull base findings on imaging that are not consistent with a tumor or infection. Lotfallah et al. and Harrison et al. have described GPA presenting with skull base osteomyelitis and cranial neuropathies as well [5, 6]. In each of those cases, patients failed to respond to antibiotics. Our case, along with these other unique cases, calls for increased consideration of GPA/autoimmune workup in patients with imaging findings concerning for a lateral skull base infection in the absence of clinical manifestations and classic laboratory markers of a serious infection.

## 4. Conclusion

Due to the wide variation of GPA presentations and the relatively rare scenario of primary skull base involvement, GPA with nonclassic findings can pose a diagnostic challenge. Available treatments can be highly effective for GPA, and it is important for healthcare teams to be aware of the variable clinical manifestations of GPA to avoid diagnostic delays and unnecessary medical treatments. For patients presenting with nasal septal perforations, severe nasal crusting, and additional nonspecific findings, evaluating physicians should consider a vasculitic etiology, such as GPA. Early autoimmune work-up should be considered in patients with atypical otologic symptoms and imaging suggestive of skull base pathology when GPA remains on the differential, especially in the absence of infectious signs.

## Data Availability

Data sharing is not applicable to this article as no new data were created or analyzed in this study.
